# Treating Glaucoma in Intellectually Disabled Patients: Novel Criteria for Choosing Surgical Candidates

**DOI:** 10.1155/joph/9752978

**Published:** 2025-06-09

**Authors:** Mitchell G. Nash, Joseph G. Parrish, David Fleischman

**Affiliations:** ^1^The Eye Center of Oak Ridge, Oak Ridge, Tennessee, USA; ^2^Department of Ophthalmology, University of Florida, Gainesville, Florida, USA; ^3^Department of Ophthalmology, University of North Carolina at Chapel Hill, Chapel Hill, North Carolina, USA

**Keywords:** Down syndrome, glaucoma, glaucoma surgery, intellectual disability, ophthalmology

## Abstract

Patients with intellectual disabilities (IDs) have been associated with having a higher frequency of ocular pathologies, including glaucoma. However, despite this association, there is little guidance in the literature pertaining to patient management or outcomes after glaucoma surgery. Literature review of the management of surgical eye conditions in ID patients provides historical considerations in treatment of these patients and educates the community of providers caring for this population. Using these data, we propose a novel set of criteria for selecting which patients with ID and glaucoma should be offered glaucoma surgery.

## 1. Introduction

Glaucoma is a common, blinding eye disease characterized by optic nerve atrophy and irreversible visual field loss [1]. Patients with intellectual disabilities (IDs) have been associated with having a higher frequency of ocular pathologies, including glaucoma [2–5]. The only treatment known to be effective for glaucoma is lowering the intraocular pressure (IOP) [1]. This is performed with topical drops, oral medications, laser, and/or incisional surgical interventions. Topical drops are first-line treatment in most cases of glaucoma [1, 6]. Surgical interventions are considered for further lowering eye pressure when vision loss progresses or the eye continues to sustain damage from glaucoma despite maximum medical therapy [6]. Surgical intervention could also be considered in cases of nonadherence to long-term topical therapies due to patient intolerance or for other reasons [7].

There is literature supporting evidence of good surgical outcomes in treating other eye disorders in intellectually disabled patients, such as keratoconus and cataracts [8–12]. However, despite the known association with glaucoma and IDs, there is little guidance in the literature pertaining to safe practices and outcomes of glaucoma surgical care. Thus, we set out to better understand the unique challenges of treating patients that have both ID and glaucoma through a literature review, while also reviewing our institution's own practices in providing glaucoma care to patients with varying severity of ID. In doing so, the secondary aim is to educate health professionals that specialize in treating patients with ID regarding these challenges. This article further explores the considerations for surgical management of glaucoma in patients with ID with a review of the literature.

## 2. Methods

### 2.1. Case Series

A retrospective interventional case series of all patients with ID with any diagnosis of glaucoma or ocular hypertension was performed using ICD9 and ICD10 codes as listed in the Supporting Section. The charts were reviewed for patients' severity of ID, extent of glaucoma, interventions performed, and complications. All eligible patients with a diagnosis of ID and related conditions who have been seen at the UNC Eye Center between 2004 and 2017 and carry a diagnosis of ocular hypertension or glaucoma were included in this study. Data were recorded for patient age, average duration of follow-up through the end of the study period, patient race, severity of ID, glaucoma onset type (congenital/childhood vs. adult onset), surgical history, reintervention rates, and postsurgical medical therapy needs.

### 2.2. Literature Review

A comprehensive literature search was performed through PubMed. MeSH terms were applied during the search strategy as follows: “((“ophthalmologic surgical procedures” [MeSH Major Topic]) OR (“ophthalmology” [MeSH Major Topic])) AND ((“intellectual disability” [MeSH Major Topic]) OR (“down syndrome” [MeSH Major Topic])).” Narrowing the results to the past 35 years of publications yielded 39 articles, five of which were relevant to this study.

## 3. Results

### 3.1. Case Series

Retrospective review identified 114 patients that fit the entry criteria for the study. Average patient age at initial visit was 28.3 ± 24.2 years. Patients were followed for an average of 7.5 ± 6.1 years. Of the population studied, 55 (48.2%) patients were non-Hispanic white, 48 (42.1%) were non-Hispanic black, 6 (5.3%) were Hispanic, and none (0%) were Asian. For five (4.4%) patients, race was other or unknown. The grading of ID was noted by ICD codes. Zero patients were diagnosed with mild ID, five (4.4%) with moderate ID, 12 (10.5%) with severe ID, and 12 (10.5%) with profound ID. The severity of ID for the majority of the patients (*n* = 85) was unspecified.

The average follow-up period for patients who underwent glaucoma surgery was 3.5 years, while for those who received cyclodiode surgery, it was 2.8 years. Reintervention was required in 20% of patients, with 15% undergoing a second procedure and 5% requiring three or more surgical interventions. At the final follow-up visit, 80% of surgically treated patients maintained stable IOP without adjunctive medical therapy, while 20% required additional medical therapy.

Patients undergoing treatment fell under 10 different categories of intervention as described in Table 1. Of the 114 patients in the study, 53 (46.5%) received nonsurgical intervention with medication, 45 (39.5%) underwent varying levels of surgical intervention, 6 (5.3%) underwent observation, and 10 (8.8%) were lost to follow-up. The most common surgical treatment was placement of a tube shunt (*N* = 18 eyes, 7.9% total eyes, 29.5% of surgical interventions). Cyclophotocoagulation was the second most common procedure (*N* = 10 eyes, 4.4% of eyes, 16.4% of surgical interventions). Lensectomies were typically performed for cataract extraction, although at least 2 patients received lensectomies for angle closure in the absence of actual or presumed changes in activities of daily living or quality of life pertaining to decreased vision. Two patients underwent cataract extraction without lens implantation due to visual significance, angle closure, and patient-related factors such as profound self-trauma. Enucleation was performed for three patients (2.6%) and four eyes (1.8%) as a terminal treatment. One enucleation was performed for a blind, painful eye. One was for globe rupture secondary to external trauma by another ID individual in a patient being cared for at an institution. One patient received bilateral enucleation, due to significant self-induced injuries, although the patient had bilateral light perception. This was performed after much ethical consideration to improve quality of life of patient and caregivers. Rates of sedated exams, exams under anesthesia (EUAs), and rates and type of interventions were also recorded by severity of ID as found in Table 2.

In our cohort, 25% of the patients were identified with congenital/childhood-onset glaucoma, while the remaining 75% had adult-onset glaucoma. Among patients with congenital/childhood-onset glaucoma, 44.8% underwent surgery, compared to 17.7% in adult-onset cases. The rates of nonsurgical procedural intervention were 17.2% for congenital or childhood-onset and were 11.8% for adult-onset glaucoma as described in Table 3.

Of the patients in this study, 12 had mild glaucoma, 18 had moderate glaucoma, and 12 had severe glaucoma based on cup-to-disc ratio ≥ 0.8 or advanced visual field defects. For mild glaucoma, 2 patients (16.7%) had nonsurgical procedural intervention and 3 patients (25.0%) had surgery. In patients with moderate glaucoma, 4 patients (22.2%) had nonsurgical procedural intervention and 6 patients (33.3%) had surgery. For severe glaucoma, 3 patients (25.0%) had nonsurgical procedural intervention and 5 patients (41.7%) had surgery as found in Table 4.

Complications were recorded in two patients (1.8%), both resulting from self-induced injuries. These patients were receiving medications and had not undergone surgical or laser intervention. One patient (0.9%) experienced open globe rupture requiring enucleation while one other patient (0.9%) developed endophthalmitis.

Our analysis of the relationship between age and surgical intervention revealed that younger patients with congenital or childhood-onset glaucoma were more likely to undergo surgical treatment earlier in life. This decision was driven by the rapid progression of the disease in these patients and the challenges associated with long-term medical management in individuals with IDs. In contrast, older patients typically received surgical intervention when medical therapy failed to control IOP effectively. These findings suggest that age plays a critical role in the timing and necessity of surgical treatment in this patient population.

Our study also identified a strong correlation between the severity of glaucoma at the time of diagnosis and the decision to proceed with surgical intervention. Patients presenting with severe glaucoma, particularly those who faced difficulties with adhering to medical therapy due to their IDs, were more frequently selected for surgical treatment. This suggests that the severity of the disease at presentation is a significant factor in surgical decision making, as surgery often provides a more reliable means of controlling IOP in these high-risk patients.

Upon further review, we identified that a subset of patients who underwent glaucoma surgery also had concurrent or prior ocular surgeries, including cataract extraction and, in some cases, corneal transplants. The presence of these additional surgical interventions suggests that these patients had more advanced or complicated glaucoma, which required a multifaceted surgical approach. Specifically, several patients had cataract surgery either simultaneously with glaucoma surgery or in the years preceding it, highlighting the complexity of managing advanced glaucoma in patients with IDs. These additional data have been integrated into our analysis to provide a more comprehensive understanding of the surgical management of these patients.

### 3.2. Literature Review

This review highlights the high prevalence of glaucoma in adults with Down's syndrome, though treatment insights remain limited. Most relevant studies focus on patients with ID secondary to Down's syndrome, particularly in keratoconus management. These articles were further explored to determine if their findings could be applied to treatment of glaucoma in similar patient populations.

Frantz et al. [8] reported on five Down's syndrome patients undergoing penetrating keratoplasty (PK) for keratoconus, showing an 80% graft clarity rate, which is slightly lower than that in the general population. The study emphasized the importance of close observation by a single caregiver and recommended avoiding surgery in patients prone to eye rubbing or self-trauma.

Wroblewski et al. [11] expanded on this with a larger series of 18 eyes in 13 patients, noting an 85.7% graft clarity rate. The study highlighted the necessity of intensive postoperative follow-up to detect complications early and recommended increased office visits for patients with ID.

Völker-Dieben et al. [12] compared corneal graft survival between patients with and without Down's syndrome, noting higher complication rates in the Down's group. To mitigate these risks, the study proposed criteria for selecting surgical candidates, including the ability to tolerate postoperative care and the presence of a motivated caregiver.

Koller et al. [9] studied 38 eyes in 29 patients and found an 86% graft survival rate at most recent follow-up. The study underscored the importance of caregiver diligence and suggested that specialized caregiver training could further improve outcomes. The authors questioned whether the patients with ID have been undertreated given the potential benefits of PK.

Additionally, Li et al. [10] examined cataract surgery in 20 adult Down's syndrome patients, finding delayed diagnosis and treatment in this group. The study recommended annual eye exams for patients with ID to prevent such delays and emphasized the importance of timely intervention to avoid complications.

## 4. Discussion

There is a high risk of glaucoma in the intellectually disabled patient population, and unfortunately there is little guidance in the literature on how to manage these patients [5]. Given the complexity of glaucoma surgical care and its postoperative management, ophthalmologists must confront many factors prior to deciding on medical versus surgical therapy [1, 6].

### 4.1. Case Series

One aim of this study was to understand how these patients are being managed and treated at one institution, prior to implementation of these proposed criteria. We reviewed cases of 114 patients with glaucoma and ID treated at UNC Medical Center over a 14-year period. Our study found that glaucoma surgery was not uncommon in ID patients, and there was a low complication rate. Of course, there are many confounding variables here, such as the surgeon's discretion over the appropriateness for surgery on a given patient. Although there were no defined criteria for which patients received glaucoma surgery, this process is likely selected for patients most likely to succeed from surgical intervention. In order to address different severities of ID, we examined the frequencies of surgery and complications and found that surgery was performed most frequently in eyes of patients with severe ID compared to moderate and profound ID. The most notable complication from the chart review was globe trauma resulting in enucleation in a patient with profound ID. This trauma occurred in a patient who received care at an institution with other ID patients. The trauma to the patient's eye originated from another ID patient at the facility. In our cohort, 25% of the patients were identified with congenital/childhood-onset glaucoma, while the remaining 75% had adult-onset glaucoma. Surgical intervention was more frequently pursued in patients with congenital/childhood glaucoma due to the aggressive nature of the disease and the difficulty in maintaining long-term compliance with medical therapy. Adult-onset glaucoma cases were managed with a combination of medical and surgical approaches, with surgery being considered primarily when medical therapy was insufficient.

Another notable finding from this study is comparing rates of sedated exams and EUAs based on severity of ID. These exams are required when the patient is unable to cooperate with the ophthalmologic exam in clinic [13, 14]. There was a positive correlation between the use of sedated exams in clinic and the severity of ID of the patient. Upon review of the charts, no patients (0%) of the five diagnosed with moderate ID required a sedated exam while 7 of 12 (58.3%) with severe ID and 6 of 12 (50%) with profound ID required a sedated exam. A similar trend was noted with EUAs, again with an increase in the need for EUAs with increasing severity of ID. Of those with moderate ID, one patient of the five (20%) required an EUA while 5 of 12 (41.7%) with severe ID and 7 of 12 (58.3%) with profound ID required an EUA. These findings show a direct relationship between the severity of ID and the rate of EUA.

The use of these exams is necessary when ophthalmologists are unable to obtain information from the exam which may alter the management of the patient. A sedated exam can be useful if the patient has mild to moderate levels of anxiety or becomes overstimulated from the exam. Sedation can sometimes calm the patient to a level where they can be examined while retaining the ability to participate in portions of the exam which require patient directed eye movements. Sedation for these exams can be achieved with various agents such as ketamine (intravenous (IV) or intramuscular), midazolam (IV, intranasal, or oral), dexmedetomidine (IV or intranasal), and propofol (IV) [13]. EUAs are reserved for patient refusal of examination, making obtaining necessary clinical data impossible. If the patient forcibly shuts their eyes or rolls their eyes to avoid light, an EUA may be required. For these exams, patients are intubated and placed under general anesthesia [14]. The increasing rate of EUA with increasing severity of ID corresponds with how patients with severe and profound ID have a more limited capacity for cooperating with staff and understanding of the benefits of the exam. The decrease in rates of sedated exams in patients with profound ID compared to severe ID is likely due to the fact that patients with profound ID were unable to participate in the exam with sedation, and thus these patients required EUAs instead. Further, a patient who is agitated will likely hold their breath or strain, artificially or momentarily increasing their IOP at time of tonometry.

The decision for how to manage patients with glaucoma is challenging even for the general population without comorbidities [1]. Our study objective was to determine what additional considerations were necessary when treating glaucoma in patients with ID. The only treatment known to be effective for managing glaucoma is to lower the IOP, but there are many methods to accomplish this [1, 6]. These methods mostly fall into three categories: medical therapy, laser surgery, and incisional surgery [6]. Medical therapy with drops can be effective for lowering the IOP and are the most commonly used first-line therapy. When titrating medical therapy, the ophthalmologist must find a balance of the effectiveness and the side effects of the drops appropriate for each patient. The limitations of drops alone are the need for life-long compliance and increased volatility in IOP [7]. In ID, the need for lifelong drops can be a particular challenge due to the toll it can have on the patient and caregiver relationship over time.

A particular focus of the study is understanding which patients can safely receive surgical intervention to help lower their IOP. In some cases of glaucoma, the IOP remains elevated despite medical management [6]. A benefit of surgical intervention for treatment of glaucoma is lower and more stable IOP over time [15]. Better long-term control is particularly valuable in treating glaucoma in a younger patient because the disease is life-long and vision lost is impossible to recover, and many patients with ID who develop glaucoma do so at a young age [5, 16]. However, surgery is not without substantial risk [17–19]. Incisional surgery creates alternate pathways for aqueous fluid to drain from the anterior chamber. This disruption of the native anatomy leads to increased vulnerability to damage from external forces. The eye can be catastrophically injured with eye rubbing or trauma from another individual or object leaving the patient worse off than if they had never received surgical treatment [20, 21].

Glaucoma has many etiologies, and the pathogenesis can be multifactorial [1]. However, a theme common to many forms of glaucoma is an increased resistance in the outflow tract of aqueous humor from the anterior chamber of the eye, particularly at the level of the trabecular meshwork (TM) [22–25]. The embryologic origin of this area is neural crest cells [24]. In many forms of ID, there is disruption of neural crest cell development and migration. Examples include cardiac defects, craniofacial abnormalities, and thyroid defects [26]. Therefore, a theory for the increased prevalence of glaucoma in patients with IDs is presence of aberrant anatomy at the level of the TM [27, 28]. None of the medications used to treat glaucoma create lasting changes in the anatomy of the TM or other structures of aqueous outflow [1]. Therefore, these medications do not address a potential root cause of glaucoma in these individuals. Surgical intervention aims to repair the dysfunctional TM or to create a new channel for the aqueous fluid to bypass the TM. Patients with ID and glaucoma may benefit from surgical intervention because it directly addresses an underlying mechanism of the disease [15, 23, 24].

The findings of this study demonstrate high rates of surgical intervention (Table 1) both as a last resort treatment option as well as in select patients deemed to be quality candidates for invasive procedures. There were no postoperative complications among patients who underwent surgical interventions (Table 2). Thus, we conclude that surgical intervention can be safe and appropriate with proper patient selection and postoperative care.

### 4.2. Literature Review

An additional aim of the study was to review existing literature for treatment of eye disorders in patients with ID. The data included for the literature review include articles dating back to 1990. Much of the research involving surgical management of ophthalmic disease in patients with ID is dated and limited in scope. These authors used published literature to adapt the best available practice guidelines to the surgical management of glaucoma in patients with ID. All studies showed lower rates of successful outcomes in patients with ID compared to the general population [8, 9, 11, 12]. However, the gap between the two groups narrowed over time. For the corneal transplant studies, success was measured by the presence of a clear graft without graft rejection. Frantz et al. [8] noted a graft clarity rate of 80%, Wroblewski et al. [11] noted a rate of 85.7%, and Koller et al. [9] noted a rate of 86%. This compares to a graft clarity in the general population of 90%–98%. It is possible that the continued improvements of care in patients with ID are attributable to the clinical pearls shared through these research articles over time [8, 9, 11, 12].

Each article included in this review helps to educate the community of clinicians caring for those with ID in managing their comorbid ocular disorders. The recommendations from prior researchers fall into three categories presented in this review: screening, patient selection for surgery, and postoperative management.

First, Li et al. [10] placed emphasis on screening for ocular disease in individuals with ID. They recommend annual eye exams for patients with ID given the increased rate of ophthalmic disease in this population. Also, communication barriers between patients with ID and their caregivers and clinicians can lead to delays in diagnosis of these diseases. Routine visits for these patients will help to mitigate these delays [10, 29].

**Figure 1:**
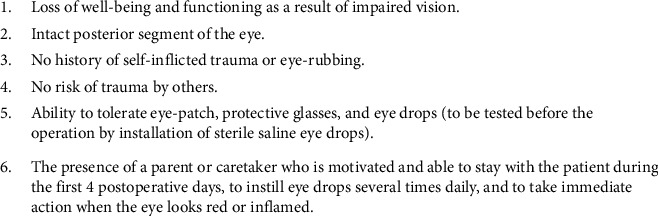
Volker-Dieben criteria for selecting patients with Down syndrome as candidates for penetrating keratoplasty surgery.

**Figure 2:**
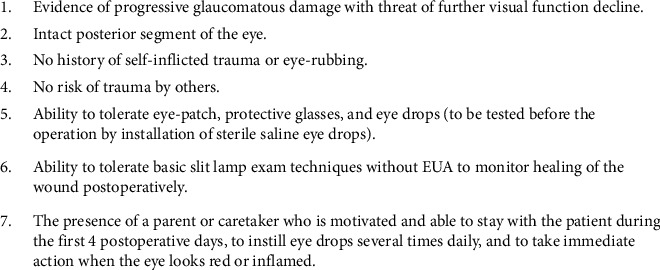
Authors proposed criteria for selecting patients with intellectual disability as candidates for glaucoma surgery.

Second, patient selection for surgical intervention must be thorough in its considerations and individualized to each patient. Wroblewski et al. [11] placed particular emphasis on the individualized preoperative evaluation. This is a logical approach as each patient with ID may be affected to varying levels and the access to resources for support may be different between patients. From the earliest study used in the review, Frantz et al. [8] singled out eye rubbing and self-traumatization as characteristics which preclude a patient from consideration for surgery. Building on this further, Völker-Dieben et al. [12] created a clear and extensive outline of characteristics which are included in Figure 1. Volker-Dieben provided the most robust guidelines for selection of patients for surgery. As these guidelines were initially created for preoperative evaluation prior to cornea surgery, we have adjusted the criteria (Figure 2) to include the recommendations of other authors in the review and to adapt it for the evaluation of glaucoma surgery. Most notably, the first criterion listed has been changed to note a decline in vision rather than reserving surgery for after the patient has a loss of functioning. This is because in corneal pathologies, vision lost can be recovered following surgery. In glaucoma, vision lost is impossible to recover [1, 6, 16, 30]. Therefore, ophthalmologists must act before the patient is incapacitated by loss of vision [30].

After acting, thorough postoperative care is essential for positive patient outcomes. Frantz et al. [8]; Wroblewski et al. [11]; and Koller et al. [9] all place emphasis on quality caregiver support in this period. Koller et al. [9] also point to evidence that specialized training of these caregivers could improve patient outcomes as well [31]. Close observation by a single caregiver allows for early diagnosis of postoperative complications [32]. These complications were most often caused by bacterial infection and trauma as reported by Volker-Dieben et al. [12]. Wroblewski et al. [11] also noted that increased frequency of postoperative clinic visits increases patient familiarity and confidence and leads to early discovery of postoperative complications. Together, these articles teach us how to screen, assess, and manage ID patients with glaucoma [8–12].

### 4.3. Surgical Technique and Follow-Up

In our patient cohort, tube shunt surgery was the most commonly performed procedure, favored for its ability to maintain structural integrity of the eyeball and reduce long-term infection risk. However, the potential for corneal complications due to anterior chamber tube placement, particularly in patients prone to eye rubbing, warrants careful consideration. On the other hand, trabeculectomy, especially when combined with mitomycin C, can weaken the sclera and increase the risk of late-onset infections. This highlights the importance of selecting the appropriate surgical technique based on individual patient characteristics, including the risk of postoperative complications and the likelihood of adherence to postoperative care.

The average follow-up period of 3.5 years was sufficient to observe the long-term outcomes of the surgeries, including the need for additional interventions or the maintenance of IOP control. During the follow-up period, 20% of the patients required additional surgical interventions beyond the initial procedure. Specifically, 15% of the patients underwent a second surgery to manage complications or further reduce IOP, while 5% required three or more surgical interventions. These findings underscore the complexity of managing glaucoma in patients with IDs, as well as the importance of careful surgical planning and follow-up.

### 4.4. Limitations

This study is not without limitations. Many younger patients with ID and glaucoma elected to receive care with a pediatric glaucoma specialist at a nearby tertiary care center. Thus, follow-up data for these patients were unavailable to these researchers. This study does not distinguish between different etiologies of glaucoma which have different algorithmic managements. Measures of outcome in IOP and vision were not included, and future investigations should focus on this. Drugs and dosage for sedation were not recorded for sedated exams. The criteria proposed for choosing surgical candidates was not recorded in an objective way at the time of treatment. Some of the proposed criteria require subjective interpretation. Syndromic disorders and classifications of disability were not recorded in data collection. No formal IQ or ID testing was performed by these researchers; thus, the data may be underreported due to potential for underdiagnosis of ID. The severity of ID was not charted for most patients in the study, and no patients in the study had a classification of mild ID.

### 4.5. Conclusion

The criteria set forth serve as a guide to caregivers in all fields that treat patients with glaucoma and ID. In order to assist in care of this population, primary care and ID specialists should provide thorough documentation of the functional status of patients in referrals to ophthalmologists or in preprocedure treatment planning. In turn, ophthalmologist should illicit this information from the primary providers as well as review the proposed criteria with the patient and caregiver in treatment planning. Ophthalmologists should not exclude surgery in treatment of these patients if they fit these criteria. Koller et al.'s study was published 21 years after Volker-Dieben et al.'s study [9] [12]. There is evidence of heavy influence of Volker-Dieben et al.'s work in its review. Koller et al.'s sttudy has the largest case series, is most recent, and has supeiror outcomes compared to the rest of the published data. This supports the notion that we can safely treat surgical eye disorders in ID patients through implementation of explicit and thorough criteria. Interestingly, in our own center's review of postoperative follow-up, it was revealed that 80% of patients who underwent surgical intervention achieved stable IOP without the need for additional medical therapy. In the remaining 20%, adjunctive medical therapy was required to maintain IOP control. These findings suggest that while surgery can significantly and safely reduce the burden of medical therapy, careful monitoring and follow-up are essential to ensure long-term success. Future studies should collect more data on IOP and etiology of glaucoma to better understand which surgical treatment modalities provide superior efficacy. Eventually, this population should have dedicated RCTs in order to create guidelines for best practice patterns for glaucoma in ID.

Our study underscores the importance of considering both age and the severity of glaucoma when deciding on surgical intervention for patients with IDs. Additionally, the presence of other ocular conditions requiring surgery, such as cataracts or corneal disease, may indicate more severe glaucoma that necessitates surgical management. These factors should be carefully weighed to optimize outcomes. Finally, our findings highlight not only the initial success of surgical interventions in managing glaucoma in patients with IDs but also the necessity of ongoing follow-up and the potential need for additional surgical procedures. The extended follow-up periods and the number of reoperations provide critical insights into the long-term management and challenges of treating this vulnerable patient population.

## Figures and Tables

**Table 1:** Summary of medical and surgical interventions.

Intervention	Patients	Eyes
Drops	53	83
Tube shunt	13	18
Trabeculectomy	4	5
Goniotomy/trabeculotomy	6	8
Goniosynechialysis	1	1
Selective laser trabeculoplasty	3	5
Cyclophotocoagulation	8	10
Endoscopic cyclophotocoagulation	1	1
Lensectomy without IOL placement	6	9
Enucleation	3	4
Observed	6	12
Lost to follow-up	10	20
Total	114	176

**Table 2:** Intervention profile by severity of intellectual disability.

	Moderate ID	Severe ID	Profound ID	Total (including unspecified ID severity)
Number of patients	5	12	12	114
Received sedated exam	0 patients	7 patients (58.3%)	6 patients (50%)	18
EUA	1 patient (20%)	5 patients (41.7%)	7 patients (58.3%)	34
Receiving drops at last follow-up	4 eyes (40%)	14 eyes (58.3%)	15 eyes (62.5%)	53
Procedures	CPC (1 eye) (10%)	LPI (1 eye) (4.2%)	CPC (3 eyes) (12.5%)	12
Surgeries	0	Goniotomy (2 eyes) Trabeculectomy (2 eyes) Tube shunt (2 eyes) PK (1 eye) Enucleation (1 eye)	Tube shunt (3 eyes) PK (1 eye) Enucleation (1 eye)	33

**Table 3:** Intervention profile by congenital/childhood versus adult-onset glaucoma.

	Congenital/childhood-onset glaucoma	Adult-onset glaucoma
Number of patients	29	85
Procedures	5 (2 SLT, 2 LPI, 1 CPC)	10 (4 SLT, 4 LPI, 2 CPC)
Surgeries	13 (3 goniotomy) (2 trabeculectomy) (6 tube shunt) (2 enucleation)	15 (2 goniotomy) (4 trabeculectomy) (6 tube shunt) (3 enucleation)

**Table 4:** Intervention profile by severity of glaucoma.

Severity (number)	Procedures	Surgeries
Mild (12)	2 (16.7%)	3 (25.0%)
Moderate (18)	4 (22.2%)	6 (33.3%)
Severe (12)	3 (25.0%)	5 (41.7%)

## Data Availability

The data supporting the findings of this study are available upon reasonable request from the corresponding authors.

## Supporting Information

The supporting document includes a complete list of ICD-9 and ICD-10 diagnostic codes used to identify patients with glaucoma or ocular hypertension included in the study.
