# Supplementary material for: Treating Glaucoma in Intellectually Disabled Patients: Novel Criteria for Choosing Surgical Candidates
Source: J Ophthalmol. 2025 Jun 9;2025:9752978. doi: 10.1155/joph/9752978 (PMC12170069; doi:10.1155/joph/9752978)
Supplement: Supporting Information — Additional supporting information can be found online in the Supporting Information section. [file 9752978.f1.docx]

Intellectual Disability/Mental retardation

ICD-9

317.*

318.*

319.*

ICD-10:

F70.*

F71.*

F72.*

F73.*

F79.*

Specific delay in development

ICD-9

315.*

ICD-10:

F81.*

F80.1

F80.2

H93.25

F80.81

F80.0

F80.89

F82

F88

F81.9

F89

Autism / Aspergers / other specified pervasive

ICD-9

299.*

ICD-10:

F84.*

Chrosomal abnormalities: (includes Down’s syndrome, Patau syndrome, Edwards syndrome)

ICD-9

758.*

ICD-10:

Q90.*

Q91.7

Q91.3

Q93.4

Q93.81

Q93.88

Q93.3

Q93.7

Q93.89

Q92.8

Q99.8

Q99.9

Glaucoma

ICD-9

365.* (includes glaucoma suspects, ocular hypertension)

ICD-10:

H40.*

Q150.*

H42.*
